# Replication of Association between Schizophrenia and Chromosome 6p21-6p22.1 Polymorphisms in Chinese Han Population

**DOI:** 10.1371/journal.pone.0056732

**Published:** 2013-02-21

**Authors:** Yang Zhang, Tianlan Lu, Hao Yan, Yanyan Ruan, Lifang Wang, Dai Zhang, Weihua Yue, Lin Lu

**Affiliations:** 1 National Institute on Drug Dependence, Peking University, Beijing, China; 2 Institute of Mental Health, Peking University, Beijing, China; 3 Key Laboratory of Mental Health, Ministry of Health (Peking University), Beijing, China; Kunming Institute of Zoology, Chinese Academy of Sciences, China

## Abstract

Chromosome 6p21-p22.1, spanning the extended major histocompatibility complex (MHC) region, is a highly polymorphic, gene-dense region. It has been identified as a susceptibility locus of schizophrenia in Europeans, Japanese, and Chinese. In our previous two-stage genome-wide association study (GWAS), polymorphisms of zinc finger with KRAB and SCAN domains 4 (*ZKSCAN4*), nuclear factor-κB-activating protein-like (*NKAPL*), and piggyBac transposable element derived 1 (*PGBD1*), localized to chromosome 6p21-p22.1, were strongly associated with schizophrenia. To further investigate the association between polymorphisms at this locus and schizophrenia in the Chinese Han population, we selected eight other single-nucleotide polymorphisms (SNPs) distributed in or near these genes for a case-control association study in an independent sample of 902 cases and 1,091 healthy controls in an attempt to replicate the GWAS results. Four of these eight SNPs (rs12214383, rs1150724, rs3800324, and rs1997660) displayed a nominal difference in allele frequencies between the case and control groups. The association between two of these SNPs and schizophrenia were significant even after Bonferroni correction (rs12000: allele A>G, *P = *2.50E-04, odds ratio [OR] = 1.27, 95% confidence interval [CI] = 1.12–1.45; rs1150722: allele C>T, *P* = 4.28E-05, OR = 0.55, 95% CI = 0.41–0.73). Haplotype ATTGACGC, comprising these eight SNPs (rs2235359, rs2185955, rs12214383, rs12000, rs1150724, rs1150722, rs3800324, and rs1997660), was significantly associated with schizophrenia (*P* = 6.60E-05). We also performed a combined study of this replication sample and the first-stage GWAS sample. The combined study revealed that rs12000 and rs1150722 were still strongly associated with schizophrenia (rs12000: allele G>A, *P*_combined_ *= *0.0019, OR = 0.81; rs1150722: allele G>A, *P*_combined_ *= *3.00E-04, OR = 0.61). These results support our findings that locus 6p21-p22.1 is significantly associated with schizophrenia in the Chinese Han population and encourage further studies of the functions of these genetic factors.

## Introduction

Schizophrenia is a psychiatric disorder with a worldwide prevalence of up to 1% and highly heritable factors. It is characterized by disturbances in thinking, emotion, cognition, and social function, including hallucinations, delusions, and apathy. Prenatal immune activation appears to be a risk factor that may play an etiological role in schizophrenia [1] and may also influence the neurodevelopment process and neurotransmitter-dependent functions [2].

Many previous genetic studies reported an association between schizophrenia and locus 6p22-24, which includes the human major histocompatibility complex (MHC) region [3]–[7]. Although some studies have found negative results [8]–[13], this locus, especially the MHC region, is still a high susceptibility factor in schizophrenia [14]. Classical MHC gene products, including MHC class I and II molecules, have become known as leukocyte antigens, and their primary function is to provide protection against pathogens. Compared with classic MHC molecules, the histone supercluster, the Zinc-finger supercluster, the heat shock cluster, and other immune-related/unrelated genes have been found in the extended MHC region [15].

In recent years, genome-wide association studies (GWASs) have been a powerful and efficient approach to identifying genetic variants that are associated with complex human diseases. The methodology of GWASs has been facilitated by technological advances that enable the simultaneous and cost-effective analysis of large numbers of genetic markers in the human genome [16], [17]. GWASs of schizophrenia have identified several genes within the extended MHC region (6p21.2-p22.3) as a susceptibility locus in European [18]–[20] and Japanese [21] individuals. Additionally, our earlier two-stage GWAS also found that three single-nucleotide polymorphisms (SNPs; i.e., rs1233710 in *ZKSCAN4*, rs1635 in *NKAPL*, and rs2142731 in *PGBD1*) within this locus had a strong association with schizophrenia in a Chinese Han population [22]. These three SNPs span 35 kb and may not fully indicate chromosome 6p21-p22.1 (rs1233710 and rs1635 had the most linkage disequilibrium [LD]). To further verify our evidence of an association, we selected eight other SNPs located in or adjacent to these three genes and performed another independent case-control association study in Chinese Han subjects who are unrelated to the previous GWAS samples. We also performed a combined study using this replication sample and the first-stage GWAS sample. Because no identified functions have been reported for these three proteins, verification of the previous associations may also confirm the importance of these genetic factors and encourage further research.

## Materials and Methods

### Ethics Statement

Approval for this study was obtained from the Ethical Committee of the Institute of Mental Health, Peking University. All of the participants were adults. The objective and procedures of this study were explained to all of the subjects and patient’s guardians, and written consent was obtained. Healthy control subjects signed informed consent forms themselves. While all of the patients’ guardians signed informed consent forms on the behalf of the patients. If the patients were within a stable period according to the clinical features and could understand the consent, they also double-signed the consent forms themselves, accompanied by their guardians. Otherwise, guardians solely consented on the behalf of patients whose capacity to consent was compromised.

### Subjects

In the present replication study, 902 unrelated schizophrenia patients (484 males and 418 females; mean age, 39±7 years) and 1,091 healthy controls (559 males and 532 females; mean age, 45±9 years) were recruited. All of these participants were Chinese Han descendants and unrelated to the previous GWAS samples. The patients were recruited from the Institute of Mental Health, Peking University, Beijing, China. The clinical diagnosis was made according to the *Diagnostic and Statistical Manual of Mental Disorders*, 4th edition (DSM-IV). None of the participants had severe medical complications. Healthy controls were selected by psychiatrists using a simple non-structured interview, excluding individuals with histories of mental health problems or neurological diseases. Both the schizophrenia patients and healthy controls were Chinese Han and lived in the same area of northern China. The two groups were matched for gender and age. In the combined study, the subjects from the first-stage study were exactly the same as those we described previously [22].

### SNP Selection

We consulted the dbSNP (http://www.ncbi.nlm.nih.gov/snp; accessed October 12, 2012) and HapMap (release #24, CHB; http://hapmap.ncbi.nlm.nih.gov/; accessed October 12, 2012) databases and determined the LD block using the criterion of *D*′ >0.80 and Haploview version 4.0 [23]. The locus that we determined spanned 63 kb and covered four genes (from telomere to centromere: *ZKSCAN4*, *NKAPL*, *ZNF187*, and *PGBD1*) and nearby regions. Single-nucleotide polymorphisms with minor allele frequencies (MAFs) <5% were excluded from the genetic analysis according to the HapMap CHB population. Therefore, no SNPs were chosen in the *ZNF187* gene. Additionally, for the combined analysis, our GWAS data were consulted. Single-nucleotide polymorphisms with *P* values that did not reach genome-wide significance but were <0.05 were considered in this study [22]. Finally, eight SNPs were selected, and their positions are shown in Table 1. All eight SNPs were tag SNPs and displayed strong associations with schizophrenia in our previous GWAS. Three of the SNPs (rs12000, rs3800324, and rs1997660) were located in exons, and their alleles were recognized as missense mutations.

**Table 1:** Genotype and allele frequencies of eight SNPs and results of comparison of schizophrenia case and control groups.

								*χ^2^* (*df* = 2)			*χ^2^* (*df* = 1)	
Gene	Position	SNP	*N* c	Genotype a			HWE (*P*)	*P* value b	Allele a		*P* value b	OR (95% CI)
*ZKSCAN4*	28323620	rs2235359		AA	AC	CC			A	C		
		Cases	898	718 (0.800)	163 (0.182)	17 (0.019)	0.034	2.845	1599 (0.890)	197 (0.110)	1.169	0.89 (0.73–1.10)
		Controls	1089	884 (0.812)	194 (0.178)	11 (0.010)	0.922	0.241	1962 (0.901)	216 (0.099)	0.280	
*ZKSCAN4*	28330959	rs2185955		CC	CT	TT			C	T		
		Cases	895	20 (0.022)	159 (0.178)	716 (0.800)	0.003	3.312	199 (0.111)	1591 (0.889)	1.394	1.13 (0.92–1.39)
		Controls	1089	13 (0.012)	191 (0.175)	885 (0.813)	0.459	0.191	217 (0.100)	1961 (0.900)	0.238	
*NKAPL*	28331710	rs12214383		CC	CT	TT			C	T		
		Cases	891	173 (0.194)	456 (0.512)	262 (0.294)	0.312	4.773	802 (0.450)	980 (0.550)	4.392	1.14 (1.01–1.30)
		Controls	1089	187 (0.172)	534 (0.490)	368 (0.338)	0.777	0.092	908 (0.417)	1270 (0.583)	0.036	
*NKAPL*	28335415	rs12000		AA	AG	GG			A	G		
		Cases	882	353 (0.400)	413 (0.468)	116 (0.132)	0.780	13.737	1119 (0.634)	645 (0.366)	13.424	1.27 (1.12–1.45)
		Controls	1086	355 (0.327)	543 (0.500)	188 (0.173)	0.425	0.001	1253 (0.577)	919 (0.423)	2.50E-04	
*PGBD1*	28358215	rs1150724		AA	AG	GG			A	G		
		Cases	891	270 (0.303)	461 (0.517)	160 (0.180)	0.129	6.829	1001 (0.562)	781 (0.438)	5.462	0.86 (0.76–0.98)
		Controls	1091	391 (0.358)	524 (0.480)	176 (0.161)	0.984	0.033	1306 (0.599)	876 (0.401)	0.019	
*PGBD1*	28361511	rs1150722		CC+CT ^d^		TT			C	T		
		Cases	895	3+63 (0.003+0.070)		829 (0.926)	0.132	16.218^d^	69 (0.039)	1721 (0.961)	16.765	0.55 (0.41–0.73)
		Controls	1091	8+133 (0.007+0.122)		950 (0.871)	0.166	5.64E-05	149 (0.068)	2033 (0.932)	4.28E-05	
*PGBD1*	28372660	rs3800324		AA	AG	GG			A	G		
		Cases	891	49 (0.055)	336 (0.377)	506 (0.568)	0.484	5.273	434 (0.244)	1348 (0.756)	5.114	0.85 (0.73–0.98)
		Controls	1088	80 (0.074)	439 (0.403)	569 (0.523)	0.710	0.072	599 (0.275)	1577 (0.725)	0.024	
*PGBD1*	28377642	rs1997660		CC	CT	TT			C	T		
		Cases	889	268 (0.301)	455 (0.512)	166 (0.187)	0.266	6.107	991 (0.557)	787 (0.443)	5.420	0.86 (0.76–0.98)
		Controls	1089	384 (0.353)	526 (0.483)	179 (0.164)	0.960	0.047	1294 (0.594)	884 (0.406)	0.020	

a Frequencies are shown in parentheses.

b Significant *P* values (<0.00625, Bonferroni corrected α) are in bold.

c Number of samples that are well-genotyped. ^d^ CC and CT genotypes were analyzed as one group because the number of CC cases was less than five (*df* = 1).

OR, odds ratio; CI, confidence interval; *df*, degrees of freedom; HWE, Hardy-Weinberg equilibrium.

### Sample Preparation and Genotyping

Peripheral blood samples were collected from all of the subjects. Genomic DNA was extracted using the QIAamp DNA Mini Kit (Qiagen). The genotyping of denatured samples was performed using the Sequenom MassARRAY system (Sequenom iPLEX; Bioyong Technologies, Beijing, China) according to the manufacturer’s instructions. Approximately 15 ng of genomic DNA was used to genotype each sample. Locus-specific polymerase chain reaction (PCR) and detection primers were designed using MassARRAY Assay Design 3.0 (primer sequences not shown). The sample DNAs were amplified by multiplex PCR reactions. Polymerase chain reaction products were then used for locus-specific single-base extension reactions. The resulting products were desalted and transferred to a 384-element SpectroCHIP array. Allele detection was performed using MALDI-TOF MS spectroscopy. The mass spectrograms were analyzed using MassARRAY TYPER. To evaluate genotyping quality, 5% of the samples were randomly selected, and the rs2185955 and rs12214383 SNPs were genotyped again. And no inconsistency was found.

### Statistical Analysis

Hardy-Weinberg equilibrium (HWE) was separately tested among the case and control groups using *χ* ^2^ goodness-of-fit tests with one degree of freedom (*df*). The Pearson *χ* ^2^-test was used to compare the categorical variable gender, and Student’s *t*-test was used for the continuous variable age. All of the statistical analyses were performed using SPSS 17 software. The pairwise LD analysis was applied to detect inter-marker relationships using the *D*′ value. This and the case-control association analyses were performed using Haploview version 4.0 [23] and PLINK version 1.07 [24]. Odds ratios (ORs) and 95% confidence intervals (CIs) were calculated to evaluate the effects of different alleles and haplotypes. Bonferroni corrections and permutation tests were applied to correct the *P* values of alleles (corrected α = 0.05/8 = 0.00625) and haplotypes (10,000 times), respectively, to control inflation of the Type I error rate. All of the tests were two-tailed, with statistical significance of *P*<0.05. In the combined study, heterogeneity across the two samples was evaluated using the Cochran Q statistic to determine the heterogeneity statistic (*I* ^2^) and *P* value. The combined analyses were made by using RevMan version 5. If *I^2^* was less than 50% (*P*>0.05), the fixed-effect (Mantel-Haenszel) model was used to combine the results from the two different cohorts; otherwise, the random-effect (DerSimonian-Laird) model was used [25], [26].

## Results

All of the eight SNPs we selected showed MAFs greater than 5% in our samples. The genotype distributions of the eight SNPs in the control group did not show significant deviations from HWE (Table 1). No significant differences in age or gender distributions were found between the case and control samples. The genotypes and allele frequencies of the eight SNPs are shown in Table 1. The rs2235359 and rs2185955 SNPs did not show significant differences in allele frequencies between the case and control groups. Four SNPs (rs12214383, rs1150724, rs3800324, and rs1997660) showed nominal differences in allele frequencies that disappeared after Bonferroni corrections. Significant differences remained for rs12000 and rs1150722 after the corrections (rs12000: allele A>G, *P = *2.50E-04, OR = 1.27, 95% CI = 1.12–1.45; rs1150722: allele C>T, *P* = 4.28E-05, OR = 0.55, 95% CI = 0.41–0.73).

**Figure 1:**
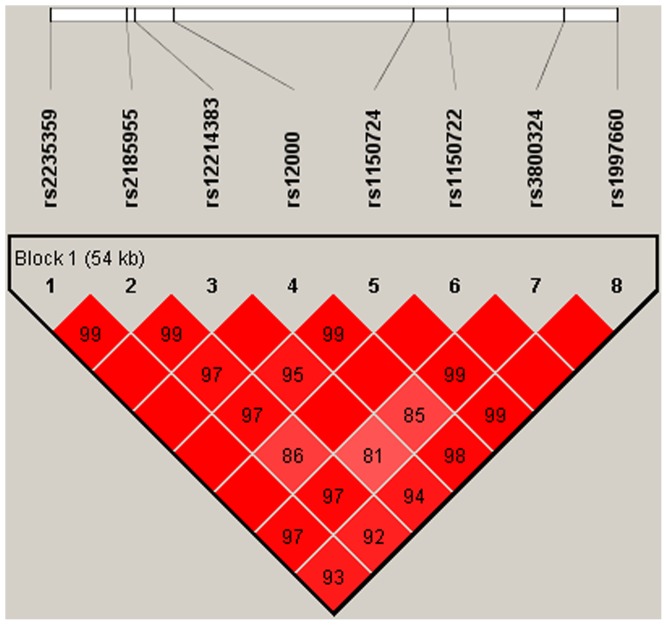
The linkage disequilibrium (LD) block structure consisted of the eight SNPs located in *ZKSCAN4*, *NKAPL*, and *PGBD1* genes. The LD pattern was derived from the combined group (i.e., both case and healthy control subjects). The LD block was defined by a *D*′ value threshold of 0.8. The color scale ranges from red to white (color intensity decreases with decreasing *D*′ value). This locus was identified as one block, and the plot was generated by Haploview.

Linkage disequilibrium was computed between every two SNPs to further analyze the haplotype structure. Fig. 1 shows the LD plot constructed using the eight SNPs. The *D*′ value of each combination was >0.8 using the combined case and control group. Therefore, we used the LD block that consisted of these eight inner-markers in the haplotype analysis (Table 2). Haplotypes ATCAGTGT and ATTGATAC showed nominal differences between the patient and control groups. ATTGACGC is a protective haplotype that is highly associated with schizophrenia (*P* = 6.60E-05, OR = 0.551, 95% CI = 0.410–0.741). This result was still significant after the 10,000-time permutation tests. Table S1 shows the results of haplotype-based association test using the same eight SNPs based on the previous GWAS dataset. The results of the present study and previous GWAS were highly consistent.

**Table 2:** Haplotype results of the entire block for the case–control association studies.

						Global	
Haplotype	Case a	Control a	*χ^2^*	*P* value b	OR (95% CI)	*χ^2^*	*P* value b
ATCAGTGT	738.31 (0.429)	846.74 (0.391)	5.863	**0.0155**	1.175 (1.031–1.340)	26.236	**2.84E-05**
ATTAATGC	282.97 (0.165)	320.94 (0.148)	1.922	0.1657	1.132 (0.950–1.348)		
ATTGACGC	67.00 (0.039)	148.00 (0.068)	15.942	**6.60E-05**	0.551 (0.410–0.741)		
ATTGATAC	372.85 (0.217)	545.79 (0.252)	6.786	**0.0092**	0.818 (0.703–0.952)		
CCTGATGC	185.02 (0.108)	208.99 (0.096)	1.270	0.2599	1.128 (0.915–1.391)		

a Frequencies are shown in parentheses.

b Significant *P* values (<0.05) are in bold.

OR, odds ratio; CI, confidence interval.

Generally, *I* ^2^<30% signifies no heterogeneity, *I* ^2^ = 30–50% signifies moderate heterogeneity, and *I* ^2^>50% indicates strong heterogeneity. In our combined analysis, the maximum *I* ^2^ was 32.6% (Table 3). Therefore, we presumed that no heterogeneity existed between these two samples and used the fixed-effect (Mantel-Haenszel) model to combine the results from the two different cohorts [25], [26]. In the combined analysis, rs12000 and rs1150722 still had strong associations with schizophrenia (rs12000: allele G>A, *P*_combined_ = 0.0019, OR = 0.81; rs1150722: allele G>A, *P*_combined_ = 3.00E-04, OR = 0.61), and significance remained even after Bonferroni correction.

**Table 3:** Combined study of replication and first-stage GWAS samples.

		Replication study (902 cases, 1091 controls)				First-stage GWAS (746 cases, 1599 controls)						
		MAF				MAF				Combined analysis		
SNP	Alleles a	Case	Control	*P* value	OR	Case	Control	*P* value	OR	*P*_combined_ b	OR (95% CI)	*I* ^2^ (%)
rs2235359	C/A	0.110	0.099	0.280	1.12	0.099	0.0757	0.006581	1.345	0.6773	1.22 (1.05–1.41)	32.6
rs2185955	G/A	0.111	0.100	0.238	1.13	0.099	0.0754	0.005839	1.351	0.6221	1.23 (1.06–1.42)	24.9
rs12214383	G/A	0.450	0.417	0.036	1.14	0.469	0.426	0.005327	1.192	0.1721	1.17 (1.07–1.28)	0
rs12000	G/A	0.366	0.423	2.50E-04	0.79	0.363	0.406	0.004405	0.8316	**0.0019**	0.81 (0.74–0.89)	0
rs1150724	G/A	0.438	0.401	0.019	1.16	0.454	0.407	0.002783	1.208	0.1061	1.19 (1.09–1.30)	0
rs1150722	G/A	0.039	0.068	4.28E-05	0.55	0.0389	0.0559	0.0131	0.6825	**3.00E-04**	0.61 (0.49–0.75)	5.3
rs3800324	A/G	0.244	0.275	0.024	0.85	0.244	0.301	5.67E-05	0.75	0.0998	0.80 (0.72–0.88)	30.0
rs1997660	A/G	0.443	0.406	0.02	1.16	0.458	0.408	0.001234	1.226	0.1105	1.19 (1.09–1.31)	0

a Minor allele/major allele.

b Significant *P*_combined_ values (<0.00625, Bonferroni corrected α) are in bold.

MAF, minor allele frequency; OR, odds ratio; CI, confidence interval.

*I* ^2^, Heterogeneity tests for Q statistics. As all of the *I^2^* less than 50% (*P*>0.05), the fixed-effect (Mantel-Haenszel) model was used to combine the results from the two different cohorts.

## Discussion

In recent years, the extended MHC region has been implicated as a main factor in schizophrenia pathogenesis, supported by GWASs of schizophrenia in different populations [18]–[22]. Association studies for markers in the human MHC region have been inconsistent, which may be attributable to heterogeneity [13], [27], [28]. The *ZKSCAN4*, *NKAPL*, and *PGBD1* genes, located in this region, are in one LD block in the Chinese Han population. In our replication study, all six SNPs within the *NKAPL* and *PGBD1* genes displayed an association with schizophrenia, but neither of the two SNPs in the *ZKSCAN4* gene showed an association. Both rs12000 and rs1150722 showed predominant significance in the present replication study and combined analysis. The haplotype analysis also indicated that the entire LD block that comprised these eight SNPs was significant in the pathogenesis of schizophrenia. Unexpectedly, Ma et al. [29] failed to validate our GWAS results in their replication study. The genetic structure of the Chinese population was investigated by two different research groups, demonstrating a population substructure among northern, central, and southern Chinese Han populations [30], [31]. The study by Chen et al. showed a strong correlation between the genetic and geographic maps of the Chinese Han population, indicating that geographic location may be a good indicator of ancestral origin, and thus geographic matching may be a good proxy for genetic matching [31]. In our study, all of the patients and controls in both the GWAS and replication samples were determined to be of northern Chinese Han origin according to their birth places and the birth places of their parents in Hebei, Shandong, and Henan provinces. In contrast, the subjects in the study by Ma et al. were recruited from Hunan province in central China [29]. The validation failure confirmed the role of regional differences in association studies of Chinese Han populations. These results also support the notion of the high genetic heterogeneity of schizophrenia.

*ZKSCAN4*, also referred to as *ZNF307,* is recognized as a member of the zinc-finger protein family. This gene did not show an association with schizophrenia, similar to our GWAS, which may attributable to the limited sample size and deviation of the patient group from HWE (Table 1). *NKAPL* is a novel gene first reported in our GWAS with a strong association on rs1635, and its product was suggested to play a role in neurodevelopmental processes [22]. It is approximately 55% homologous to *NKAP,* the protein of which has been shown to be a transcriptional repressor of notch signaling and required for T-cell development and maturation [32], [33]. Both of these proteins have a SynMuv product domain in their *C*-terminal [34]. Similar to the rs1635 SNP, the allele of rs12000 is a missense mutation. Moreover, LD was found between rs12000 and rs1635 using the HapMap database (release #24, CHB). This evidence suggests that destruction of the structure of NKAPL may affect the development of the central nervous system. *PGBD1* has been reported to be a susceptibility gene for both schizophrenia and Alzheimer’s disease [27], [35]. It is a member of the piggyBac transposable element-derived (PGBD) gene subfamily. It is also known as *SCAND4* because of the conserved SCAN domain in the *N*-terminal of its products (ZKSCAN4 also contains this domain). The SCAN domain is one type of zinc-finger protein domain and has been found in several vertebrate proteins that contain C2H2 zinc-finger motifs, many of which may be transcription factors that play roles in cell survival and differentiation [34]. This protein-interaction domain is able to mediate the homo- and hetero-oligomerization of SCAN-containing proteins [36], [37]. Although the rs2142731 and rs1150722 SNPs are both within introns of *PGBD1*, their alleles may affect the expression of PGBD1.

Numerous other genes in the extended MHC region have been shown to be involved in the pathogenesis of schizophrenia. MICB, HLA-A, and HLA-B are classic MHC molecules that play central roles in the development of host defense and immunity [38], [39]. Tumor necrosis factor α (TNFα), located in the classic class III subregion, encodes a cytokine involved in systemic inflammation and acute-phase reactions [40], [41]. Products of the MHC region implicated in the pathogenesis of schizophrenia do not only contribute to immune responses but also have general functions in different molecular biological processes [15]. Heat shock protein 70 (HSP70) works as a protein-folding machine [42]. DDR1 is a type of receptor tyrosine kinase that promotes prosurvival effects through Notch1 activation [43]. Dysbindin (the product of DTNBP1) has been characterized as a stable component of a multi-subunit complex termed BLOC-1, which has been implicated in intracellular protein trafficking and the biogenesis of specialized organelles of the endosomal-lysosomal system [44]. NOTCH4 is a Notch family member that plays a role in various developmental processes by controlling cell fate decisions. All of their encoding genes are located in the extended MHC region and have been shown to be associated with schizophrenia [45]–[50]. Agartz et al. also reported that common sequence variants in the MHC region are associated with cerebral ventricular size in schizophrenia [51]. In summary, extended MHC molecules may participate in numerous life processes, in addition to the immune response, and may influence central nervous system development.

Psychosis comprises a set of complex mental disorders that are caused by interactions between polygenic and environment factors and diagnosed by clinical symptoms. Many genetic factors have been shown to play etiological roles in diverse mental disorders, including *PGBD1* in schizophrenia and Alzheimer’s disease [22], [35], [52] and TNFα in schizophrenia and bipolar disorder [40]. This evidence suggests that multiple assemblies of polygenic risk alleles may lead to variable phenotypic outcomes during different stages of life that are recognized as various psychotic disorders.

In conclusion, we confirmed the association between chromosome 6p21-p22.1 and schizophrenia in a northern Chinese Han population. Further research is needed to fully understand the functions of these various genetic factors and their roles in the pathogenesis of schizophrenia and other mental disorders.

## Supporting Information

Table S1 Haplotype results of the entire block derived from the first-stage GWAS sample. ^a^ Frequencies are shown in parentheses. ^b^ Significant *P* values (<0.05) are in bold. OR, odds ratio; CI, confidence interval.
(DOCX)
