# Supplementary material for: Replication of Association between Schizophrenia and Chromosome 6p21-6p22.1 Polymorphisms in Chinese Han Population
Source: PLoS One. 2013 Feb 21;8(2):e56732. doi: 10.1371/journal.pone.0056732 (PMC3578928; doi:10.1371/journal.pone.0056732)
Supplement: Table S1 — Haplotype results of the entire block derived from the first-stage GWAS sample. a Frequencies are shown in parentheses. b Significant P values (<0.05) are in bold. OR, odds ratio; CI, confidence interval. (DOCX) [file pone.0056732.s001.docx]

Table S1. Haplotype results of the entire block derived from the first-stage GWAS sample.

|  |  |  |  |  |  | **Global** | |
| --- | --- | --- | --- | --- | --- | --- | --- |
| **Haplotype** | **Case ^a^** | **Control ^a^** | ***χ^2^*** | ***P* value ^b^** | **OR (95% CI)** | *χ^2^* | *P* value ^b^ |
| ATCAGTGT | 677.87(0.442) | 1073.34(0.399) | 7.615 | **0.0058** | 1.199(1.054-1.364) | 30.651 | **3.61E-06** |
| ATTAATGC | 241.23(0.157) | 411.24(0.153) | 0.144 | 0.7043 | 1.034(0.869-1.230) |  |  |
| ATTGACGC | 61.76(0.040) | 166.88(0.062) | 9.075 | **0.0026** | 0.634(0.470-0.855) |  |  |
| ATTGATAC | 342.75(0.224) | 737.93(0.274) | 13.484 | **2.40E-04** | 0.758(0.654-0.879) |  |  |
| CCTGATGC | 150.22(0.098) | 194.94(0.072) | 8.465 | **0.0036** | 1.391(1.113-1.739) |  |  |

^a^ Frequencies are shown in parentheses. ^b^ Signiﬁcant *P* values (< 0.05) are in bold.

OR, odds ratio; CI, confidence interval.
